# In This Issue

**Published:** 2002

## STRATEGIES TO PREVENT UNDERAGE DRINKING

Although it is illegal to do so, many adolescents drink alcohol; in fact, alcohol is the most commonly used drug among young people. Accordingly, numerous strategies have been developed to prevent underage drinking. Some of those strategies are school based, whereas others target adolescents’ leisure hours. Still other approaches engage families or entire communities in the prevention efforts. Public policy approaches, such as increasing the minimum legal drinking age or reducing access to alcohol (e.g., by enforcing laws against alcohol sales to minors or by increasing alcohol prices) also have been used. In addition, several programs have combined multiple components for a more comprehensive approach. Drs. Kelli A. Komro and Traci L. Toomey review the various strategies and present evidence of their effectiveness in reducing drinking among adolescents. (pp. 5–14)

## ALCOHOL COUNTER-ADVERTISING AND THE MEDIA: A REVIEW OF RECENT RESEARCH

Advertisements for alcoholic beverages are pervasive in the U.S. culture. In response to these ads, various counter-advertising measures have been implemented. According to Drs. Gina Agostinelli and Joel W. Grube, counter-advertisements fall into two broad categories: broadcast and print messages (e.g., public service announcements) or alcohol beverage warning labels. The effectiveness of broadcast and print messages depends on numerous factors, such as the emotional appeal of the message, the credibility and likability of the source of the message, and characteristics of the targeted audience (e.g., gender, drinking level, and personality characteristics). Similarly, the effectiveness of warning labels is influenced by a variety of factors, such as the design of the label, the wording of the warnings, and audience characteristics. Drs. Agostinelli and Grube also present a theoretical model—the Elaboration Likelihood Model—to help explain differences in the effectiveness of various counter-advertisement approaches in persuading the audience to change its attitudes and behaviors. (pp. 15–21)

## THE EFFECTS OF PRICE ON ALCOHOL CONSUMPTION AND ALCOHOL-RELATED PROBLEMS

A fundamental law of economics states that as the price of a product increases, the demand for the product decreases. Accordingly, increases in the price of alcohol would be expected to result in reduced alcohol consumption, write Drs. Frank J. Chaloupka, Michael Grossman, and Henry Saffer. Although Federal tax rates have remained relatively stable over the past 50 years and the inflation-adjusted monetary price of alcohol has actually declined, researchers studying the effects of price differences on alcohol consumption and its effects have found that increases in the total price of alcohol can effectively reduce drinking and heavy drinking among youth and young adults. Similarly, higher costs of alcohol can ameliorate the adverse effects of alcohol consumption, such as drinking and driving and motor vehicle crashes, health consequences of drinking, and alcohol-related violence and other crime, in both young people and adults. (pp. 22–34)

## APPROACHING ALCOHOL PROBLEMS THROUGH LOCAL ENVIRONMENTAL INTERVENTIONS

One approach to reducing alcohol-related problems centers on community-based programs aimed at changing not the behavior of the individual drinker but the environment in which drinking occurs. Such programs may target alcohol vendors; law enforcement agencies; medical clinics and treatment facilities; and/or local social organizations, such as schools, churches, business organizations, and social clubs. Drs. Andrew J. Treno and Juliet P. Lee review several international and U.S.-based projects that have attempted to reduce alcohol-related problems through such community-based environmental approaches. Although not all study results have been conclusive, several projects have noted positive effects in reducing drunk driving and alcohol-related traffic crashes, curtailing the purchase of alcohol by underage drinkers, and reducing alcohol-related injuries. Additional research is needed, however, to determine the relative effectiveness of various measures, establish the cost-benefit ratio of such environmental approaches, and assess the effectiveness of these programs in different populations of drinkers. (pp. 35–40)

## EVALUATING THE ALCOHOL ENVIRONMENT: COMMUNITY GEOGRAPHY AND ALCOHOL PROBLEMS

Geographical tools are helping researchers gain a better understanding about how certain aspects of the environment, such as the locations of alcohol outlets, influence alcohol-related problems, such as traffic crashes and violence. Drs. Paul J. Gruenewald, Lillian Remer, and Rob Lipton discuss the use of maps and mapping, spatial analysis, spatial statistics, and geomathematics, which describe and analyze community alcohol issues and problems, with the goal of helping communities develop more effective models for reducing alcohol problems. The authors note that by evaluating both the individual and environmental influences on alcohol-related problems in a community, geographical research provides critical information for understanding the dynamics of alcohol-related problems in a community and for developing effective environmental preventive interventions. (pp. 42–48)

## THE WORKPLACE AND ALCOHOL PROBLEM PREVENTION

Because most adults at risk for alcohol problems are employed, the workplace is an obvious choice as a setting for alcohol prevention. Prevention approaches there have several advantages. For example, because employees spend a great deal of time at work, a coworker or supervisor may notice changes in an employee’s behavior that signal a developing alcohol problem. In addition, employers may use their leverage to motivate employees to seek help for an alcohol problem. Drs. Paul M. Roman and Terry C. Blum describe the advantages offered by workplace prevention and discuss the use of employee assistance programs (EAPs) as well as alcohol education, health promotion programs, and peer intervention to reduce employee alcohol problems. The authors also examine risk factors for alcohol problems that exist in the work environment, such as feeling stressed or alienated in the workplace. (pp. 49–57)

## FETAL ALCOHOL SYNDROME PREVENTION RESEARCH

Birth defects caused by fetal alcohol syndrome (FAS) and other, less-severe consequences of drinking during pregnancy are relatively common and can be prevented by abstaining from alcohol during pregnancy. Dr. Janet R. Hankin discusses various approaches aimed at preventing prenatal alcohol exposure. Some of these strategies, called universal efforts, are designed to improve the public’s knowledge of FAS through measures such as news reports, public service announcements, or alcohol beverage warning labels. In contrast, selective efforts (e.g., brief interventions delivered by a health care provider) target women in their reproductive years who consume alcohol. A third method, indicated efforts, is directed toward women who have a history of drinking during pregnancy or who have delivered a child with alcohol-related effects. Dr. Hankin reports that whereas selective and indicated prevention efforts have shown some usefulness in reducing alcohol consumption during pregnancy, universal efforts have had only a modest impact that has decreased over time. (pp. 58–65)

## LINKING SCIENCE TO POLICY: THE ROLE OF INTERNATIONAL COLLABORATIVE RESEARCH

Since the 1970s, scientists have collaborated internationally to study the effects of alcohol policies on alcohol-related problems. Such collaborations allow researchers to evaluate a broader array of policy options and incorporate more areas of expertise than generally would be possible in studies conducted in a single country. Dr. Thomas F. Babor traces the history of alcohol-related international collaborative research, highlighting important studies conducted over the past 25 years. These efforts have enhanced both the practical knowledge about the effects of various alcohol policies and the process of scientific discovery. In addition, these findings enable researchers to make some generalizations regarding the effectiveness of various prevention strategies. For example, population-based approaches affecting the drinking environment and alcohol availability appear to be more effective than measures aimed at the individual drinker. Other findings suggest that alcohol policy to date rarely is driven by scientific evidence and that policies motivated by public health values may be incompatible with the economic and political values of free trade and marketing. (pp. 66–74)

